# Beyond Helper Phage: Using "Helper Cells" to Select Peptide Affinity Ligands

**DOI:** 10.1371/journal.pone.0160940

**Published:** 2016-09-14

**Authors:** M. Lisa Phipps, Antoinetta M. Lillo, Yulin Shou, Emily N. Schmidt, Chad D. Paavola, Leslie Naranjo, Sara Bemdich, Basil I. Swanson, Andrew R. M. Bradbury, Jennifer S. Martinez

**Affiliations:** 1 Center for Integrated Nanotechnologies, Los Alamos National Laboratory, Los Alamos, NM 87545, United States of America; 2 Bioscience Division, Los Alamos National Laboratory, Los Alamos, NM 87545, United States of America; 3 Space Biosciences Division, National Aeronautics and Space Administration Ames Research Center, Moffett Field, CA 94035, United States of America; 4 Institute for Materials Science, Los Alamos National Laboratory, Los Alamos, NM 87545, United States of America; US Naval Research Laboratory, UNITED STATES

## Abstract

Peptides are important affinity ligands for microscopy, biosensing, and targeted delivery. However, because they can have low affinity for their targets, their selection from large naïve libraries can be challenging. When selecting peptidic ligands from display libraries, it is important to: 1) ensure efficient display; 2) maximize the ability to select high affinity ligands; and 3) minimize the effect of the display context on binding. The “helper cell” packaging system has been described as a tool to produce filamentous phage particles based on phagemid constructs with varying display levels, while remaining free of helper phage contamination. Here we report on the first use of this system for peptide display, including the systematic characterization and optimization of helper cells, their inefficient use in antibody display and their use in creating and selecting from a set of phage display peptide libraries. Our libraries were analyzed with unprecedented precision by standard or deep sequencing, and shown to be superior in quality than commercial gold standards. Using our helper cell libraries, we have obtained ligands recognizing *Yersinia pestis* surface antigen F1V and L-glutamine-binding periplasmic protein QBP. In the latter case, unlike any of the peptide library selections described so far, we used a combination of phage and yeast display to select intriguing peptide ligands. Based on the success of our selections we believe that peptide libraries obtained with helper cells are not only suitable, but preferable to traditional phage display libraries for selection of peptidic ligands.

## Introduction

Affinity ligands are important tools for the advancement of various biomedical technologies, such as advanced therapeutics, targeted delivery, and microscopy. While antibodies have historically been the most frequently employed affinity ligands, a number of exciting applications have emerged for peptides as affinity ligands due to their useful and unique properties. For example, peptides (mimotopes), selected for binding to soluble antibodies, have been used for the discovery of disease-related epitopes, and provide the potential for vaccine development without requiring information about the etiological agent or its antigens [1]. Peptides capable of recognizing changes in the binding region of analyte-bound antibodies have also been used in high sensitivity biosensors for the detection of small molecules [2]. Finally, their ease of chemical synthesis and amino acid modification can provide peptides with higher bioavailability and resistance to proteolysis, thereby improving their stability and performance in *in vivo* studies.

The ability to generate and screen large genetically-encoded antibody/peptide libraries, displayed on either viruses or cells, has matured over the past 25 years and is now one of the primary *in vitro* methods used to select antibody or peptide ligands to almost any antigen of interest [3]. Like antibody fragments, peptide ligands are typically selected from large filamentous phage display libraries. However, since peptides selected from naïve libraries usually have low affinities and, due to their smaller size, are more easily influenced by their context (e.g. display platform), their selection benefits from high display efficiency, library purity (i.e., lack of contaminating wild type M13 phage), and the ability to eliminate display bias. Efficiency and purity are the two main attributes addressed in the work we describe here.

**Fig 1:**
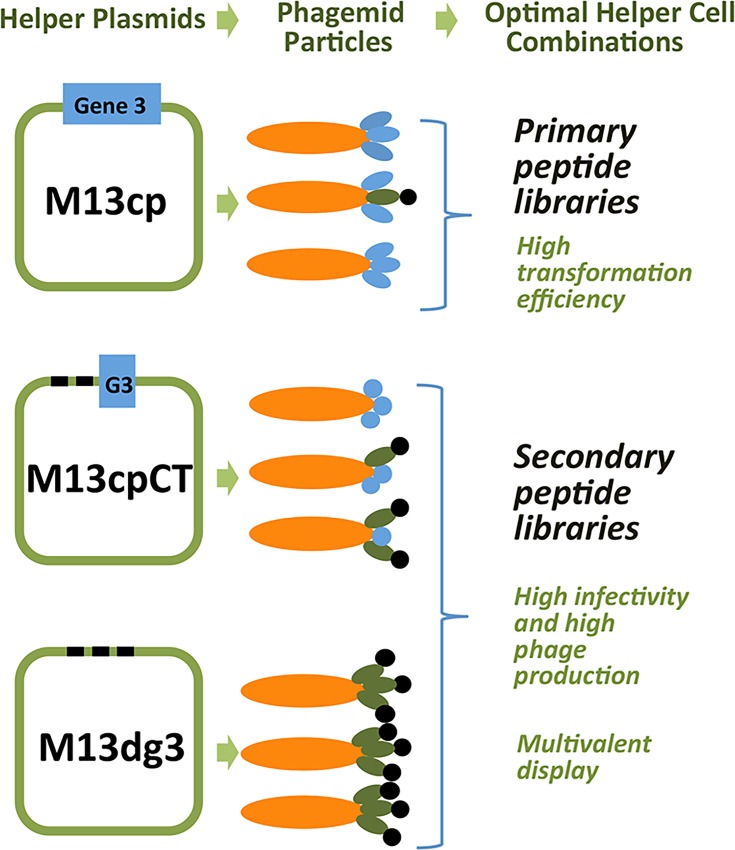
Genotypes and phenotypes of phage particles obtained with the helper cell system. Helper cells contain one of the following helper plasmids: M13cp (full-length gene 3), M13cp-CT (truncated gene 3), or M13DG3 (fully deleted gene 3). *E*. *coli* containing random-peptide-encoding phagemids and any one of the helper plasmids produce phagemid particles expressing varying levels of peptide-fused gene 3 protein. We found that M13cp cells are highly transformation efficient, making them good candidates for generating primary libraries based on the gene 3 protein display vector pDAN5 [11] while both M13cp-CT and M13DG3 cells are highly infectable, produce very high numbers of phagemid particles, and are useful to produce amplified (secondary) libraries which have the features of monovalent or multivalent display, respectively.

M13 bacteriophage is a flexible filamentous bacterial virus containing single stranded DNA packaged within a few thousand copies of p8, the major coat protein, and 3–5 copies of each of the four minor coat proteins (p3, p6, p7 and p9). By modification of the phage genome, it is possible to efficiently express polypeptides fused to phage coat proteins and build libraries of enormous complexity. The physical linkage between the phage phenotype (displayed protein/peptide) and its genotype (encoding DNA) allows the efficient selection of ligands for virtually any selector molecule [4]. Although large libraries were initially created in phage vectors, they tended to delete extraneous DNA, and it was consequently more challenging to create high complexity libraries. Early on, these problems were partially resolved by the use of phagemid display vectors, which are plasmid vectors containing a phage packaging signal and a gene encoding the displayed polypeptide-phage coat protein fusion [5]. Phagemid vectors lack all other phage protein genes, and are propagated and packaged as phage particles in bacteria only upon infection with helper phage. Helper phage is derived from M13 filamentous phage, and contains a crippled phage packaging signal, an additional origin of replication and optionally, an antibiotic resistance gene. Together, these modifications result in preferential packaging of phagemid over helper phage when both are present in a bacterial host simultaneously. Unfortunately, phagemid display libraries also have drawbacks: helper phage can sometimes dominate selections (thought to be due to a reversion of the crippled packaging signal) [6] and display is monomeric, prejudicing their use for peptides. To overcome these problems, Chasteen *et*. *al*. developed a set of helper plasmids that supply the genes necessary for packaging phage, but lack the packaging signal present in helper phage [7]. Three different helper plasmids are described: M13cp (carries a full-length p3 gene), M13cp-CT (carries a truncated p3 gene), and M13-DG3 (lacks p3 gene completely) (Fig 1). When transformed into *E*. *coli* these plasmids generate a set of helper cell lines that, upon phagemid transfection or transformation, produce phage solutions free of helper phage. Furthermore, since coat protein p3 is essential for efficient phage infectivity [5, 8–10], phage particles displaying either truncated p3 or no p3 are unable to infect unless they also contain a copy of p3 provided by the phagemid, which ensures that only phagemid with displayed proteins are propagated by infection. These helper plasmids were shown to provide different display levels of the polypeptide-phage coat fusion protein, with M13-DG3 displaying the most, M13-cp the least, and M13-cpCT in between [7]. With varying display levels, one could theoretically toggle between the higher display levels of M13-DG3 and M13cp-CT in early selection rounds to fully capture the available diversity, and the lower levels of M13cp for subsequent selections of higher affinity clones.

In phage display no more than 5 copies of a peptide or polypeptide can be displayed on each phage particle when p3 is used as the display protein. When p8 is used as the display protein, thousands of small peptides can be displayed. This is in contrast to yeast display where 30,000–100,000 polypeptides can be displayed on each cell [12, 13]. This higher display efficiency, coupled with the larger size of yeast, result in significant advantages, primarily the ability to use flow cytometry to both analyze selections and sort relevant clones based on display efficiency and affinity for the selection target. Traditional scFv display (i.e., helper phage-mediated) and yeast display have been combined as an alternative to selections based solely on phage panning [14, 15]. The scFv/yeast hybrid selection method also has the important advantage that the chance of selecting ligands with a display-context bias might be reduced. This feature could be particularly useful in selection of peptides, as we have combined here with helper cells, since small molecules like peptides are more prone to display bias.

In the work presented here we examine and refine the helper cell system by exploring different combinations of helper plasmids and commonly used *E*. *coli* strains. We used the information obtained to generate and characterize optimal peptide phage libraries displayed using the helper cell system. We chose F1V [16–19] and the glutamine-binding periplasmic protein QBP [20] as our targets for selection in order to demonstrate the power and utility of isolating peptide ligands from our helper cell libraries. F1V was chosen because ligands that bind specifically to its surface could potentially be used as reagents in biothreat sensors. L-glutamine- and apo-glutamine binding protein (QBP) was chosen because the ability to distinguish between bound and unbound proteins is valuable in sensing small molecules. When selecting QBP ligands we combined the helper cell peptide system with yeast display in the attempt to reduce the probability of selection platform bias. The results presented here show the successful use of helper cell libraries in selecting peptide ligands against targets of interest.

## Results

### Helper Cell Characterization

Our goal was to determine the best combinations of helper cell plasmids and commonly used *E*. *coli* strains to generate and amplify libraries. Three helper cell features were analyzed: transformation efficiency, ability to produce phage (phage yield) and susceptibility to phage infection (infectability). High transformation efficiency (high number of transformants) would allow us to generate larger primary libraries directly in helper cells by transformation with a fixed quantity of phagemid DNA. High phage yield would allow us to generate greater quantities of highly concentrated phage libraries with the highest possible display efficiency, improving the chances of selecting candidate binding peptides during the target-binding step of every panning cycle. Finally, high susceptibility to infection (infectability) would allow more straightforward library amplification and propagation during selections by direct infection, rather than transformation. It was previously reported that M13cp-CT and M13-DG3 helper cells produced phage particles with more efficient display than phage derived from M13cp cells [7]. Therefore we limited our search for the most infectable and most efficient phage-producing cell line to M13cp-CT and M13-DG3 helper cells. Extensive characterization of helper cells derived from various combinations of helper plasmids and *E*. *coli* strains (S1 Fig) led us to choose the high transformation efficiency cell line SS320-M13cp for cloning and production of primary libraries, and the high infectability and high phage-producing cell lines OmniMAX (Invitrogen F’ cell line #C854003) -M13cp-CT and XL1-blue-M13-DG3 for production of secondary libraries as well as amplification during panning (Fig 1).

**Fig 2:**
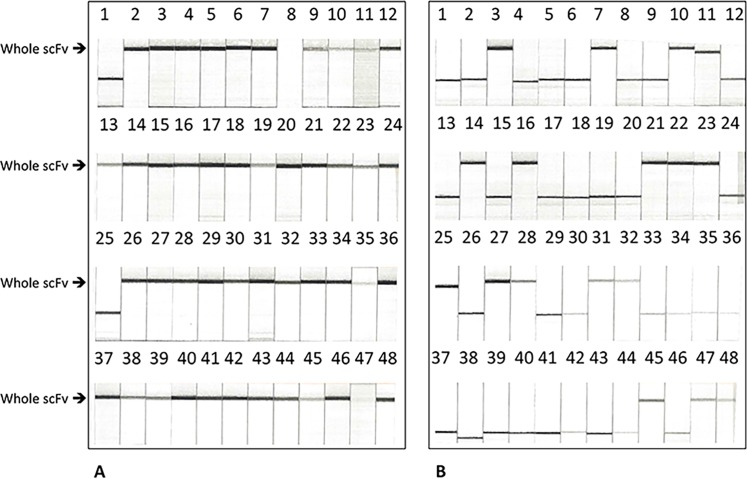
PCR analysis of scFv selections amplified with either helper phage or helper cells. Extensive characterization of helper cells derived from various combinations of helper plasmids and *E*. *coli* strains led us to choose those best suited for high transformation efficiency, high infectability and high phage-producing. Toward thorough characterization of helper cells we further investigated their utility for scFv selection. The result of the PCR and capillary electrophoresis analysis of representative number of scFv clones are shown. The DNA encoding full-length scFv correspond to the higher bands (about 800 bp), whereas the lower bands correspond to DNA encoding truncated scFv of different sizes. After the second cycle of panning, library outputs amplified with either helper phage (A) or with helper cells (B) contained approximately 4% or 66% truncated scFvs respectively. Thus, helper cells are intolerant of the scFv, leading to truncations, and instead are ideal for peptide selections.

As part of our thorough characterization of helper cells, we investigated if helper cells could be used to create scFv library uncontaminated with helper phage by transforming our primary phagemid antibody library into the M13cp cells and using the phagemid particles obtained to infect M13cp-CT derivatives of cre recombinase expressing cells, and subsequently M13cp-CT DH5alpha (Invitrogen F’ cell line #18288019) to produce the scFv display library. We quickly realized that panning of scFv libraries produced in helper cells led to enrichment of phagemid encoding truncated scFvs (Fig 2, Tables A and B in S1 File). Further examination of this phenomenon revealed that co-selection of bacteria containing both phagemid (which carries ampicillin-resistance) and helper plasmid (which carries chloramphenicol-resistance) resulted in an increase of truncated scFv (Tables C and D in S1 File). The number of truncations appeared to be related to the concentration of chloramphenicol in the selection media (Table E in S1 File). Omitting chloramphenicol reduced the level of truncation; however, since chloramphenicol is required to exert selection pressure on the helper plasmids, and the helper plasmids are required for making phage, the level of clones producing phage particles also decreased with a decreased amount of chloramphenicol. As the truncation phenomenon appeared to be due to helper cell intolerance of the scFv-fusion encoded by the phagemid vector, we speculated that the helper cell system might be better suited for producing and selecting from peptide libraries, as we have demonstrated here.

### Peptide Library Construction and Characterization

**Fig 3:**
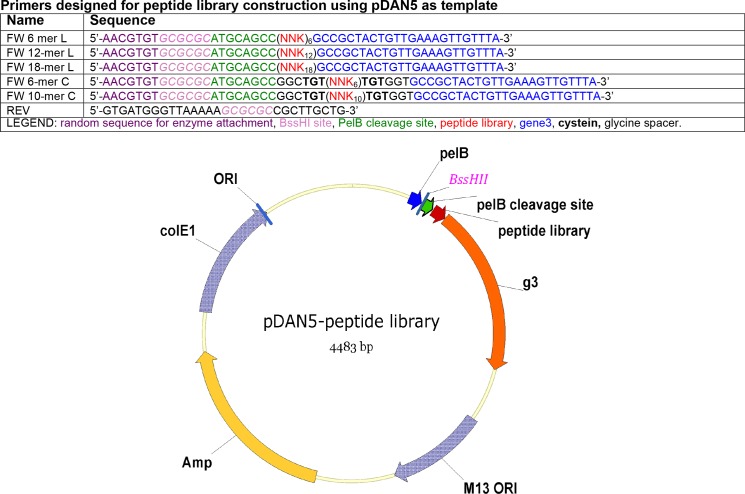
Vector map and primers used for library construction. The forward primers (FW) incorporated the different sets of random peptide sequences. The random sequences are coded by NNK, where N = A,T,C, or G and K = G or T.

The linear libraries included peptides 6, 12 or 18 amino acids long, whereas the cyclic libraries were 6 or 10 amino acids long (excluding the terminal cysteines added for cyclization). The length of our libraries was intended to bracket the full range of lengths of the complementarity determining region 3 (CDR3) found in naturally-occurring antibodies [21]. Recombinant plasmid pDAN5 [11], our standard phagemid vector, was used as template for library construction (Fig 3). The diversity of our 6-mer (linear and cyclic) and 12-mer libraries was in the 10^8^ range, the 18-mer library was in the 10^9^ range, and the 10-mer library was in the 10^8^ range. The amino acid bias (i.e. the difference between the theoretical and the observed percent of each amino acid) in our libraries was determined by sequencing, and compared to two commercially available peptide libraries from New England Biolabs (NEB), PhD-12 and PhD-C7C (see library characterization details in Table 1). Our libraries were as diverse as, if not more diverse than, these two commercial NEB libraries. Our libraries, like most commercial libraries, had fewer cysteines than theoretically expected, attributed to the tendency of unpaired disulfide bonds to interfere with phage packaging and transportation through the periplasmic membrane of the host cells. Based on the level of diversity and amino acid bias, all the libraries were determined to be suitable for selections and were panned independently against F1V. In parallel experiments a mixture of the libraries (both CT and DG3) was selected for binding to L-glutamine-binding periplasmic protein (QBP), and this was followed up with sorting by yeast display in order to identify those peptides with particular conformational preferences.

**Table 1:** Actual *vs*. theoretical percentage of each amino acid in peptide helper cell secondary libraries and commercial peptide libraries.

AA	Theor.^a^	L CPCT 6-mer	L DG3 6-mer	L CPCT 12-mer	L DG3 12-mer	L CPCT 18-mer	L DG3 18-mer	C DG3 10-mer	C CPCT 10-mer	C DG3 6-mer	C CPCT 6-mer	*NEB PhD-12* ^b^	*NEB PhD-C7C* ^b^
**F**	3.1	4.7	3.0	3.9	2.9	2.6	2.5	3.9	2.8	4.1	4.0	3.3	1.9
**P**	6.2	5.5	4.5	6.0	5.5	5.6	4.4	5.9	5.1	5.3	5.5	12.2	7.0
**M**	3.1	4.0	2.9	2.6	2.9	2.1	2.9	3.2	3.1	3.4	3.1	2.6	4.3
**G**	6.2	7.9	7.6	6.2	6.8	12.7	12.3	8.8	9.0	9.3	6.8	2.6	4.2
**A**	6.2	6.1	4.3	5.0	4.9	8.1	7.8	6.1	7.2	4.6	6.1	6.0	5.3
**V**	6.2	7.1	8.4	6.8	6.7	7.5	8.6	8.3	6.7	5.3	6.8	3.9	4.2
**L**	9.4	9.1	8.4	9.2	9.2	8.5	7.0	8.5	9.7	10.5	10.3	9.3	8.5
**I**	3.1	3.4	2.5	3.0	2.9	2.0	2.2	4.4	4.6	3.2	3.2	3.4	3.2
**S**	9.4	9.3	8.5	9.8	10.1	7.8	7.8	8.3	9.0	9.3	8.5	10.0	10.4
**T**	6.2	5.4	4.6	4.7	5.4	3.9	4.8	3.9	4.6	3.9	5.7	11.1	9.5
**Y**	3.1	3.3	3.1	3.0	4.1	2.2	2.1	3.3	1.5	2.1	3.2	3.6	3.6
**W**	3.1	3.0	3.9	3.9	2.2	4.3	4.5	2.4	4.5	4.9	4.5	2.2	2.2
**C**	3.1	0.4	1.0	1.6	1.7	1.7	1.3	1.2	2.3	1.4	1.6	0.5	0.6
**N**	3.1	3.4	1.8	3.2	2.8	2.4	2.1	2.4	1.5	2.3	3.3	4.6	4.0
**Q**	6.2	7.1	14.2	10.1	9.1	6.5	8.1	11.4	11.9	10.9	6.4	5.1	5.3
**D**	3.1	3.5	3.3	2.2	3.3	2.3	3.2	3.9	2.2	3.3	3.3	2.8	4.0
**E**	3.1	2.6	2.2	2.3	2.6	3.5	3.1	3.2	2.4	1.9	3.4	3.1	3.4
**K**	3.1	3.1	2.3	2.6	2.5	2.5	2.2	2.2	2.4	2.9	1.9	2.8	5.1
**R**	9.4	7.4	8.9	8.6	11.8	9.4	10.8	10.0	10.1	9.3	8.7	4.7	5.6
**H**	3.1	2.9	4.2	3.2	3.4	1.9	2.1	1.7	2.3	2.3	2.2	6.3	4.9

^a^ Theoretical amino acid representation was calculated according to the following formula: # codons for the amino acid indicated/total number of codons (64)*100.

^b^ Commercial peptide libraries PhD-12 (linear) and PhD-C7C (cyclic) from NEB.

Pink cells = lower percentage than theoretical.

Green cells = higher percentage than theoretical

### Selection of F1V-binding Peptides

**Fig 4:**
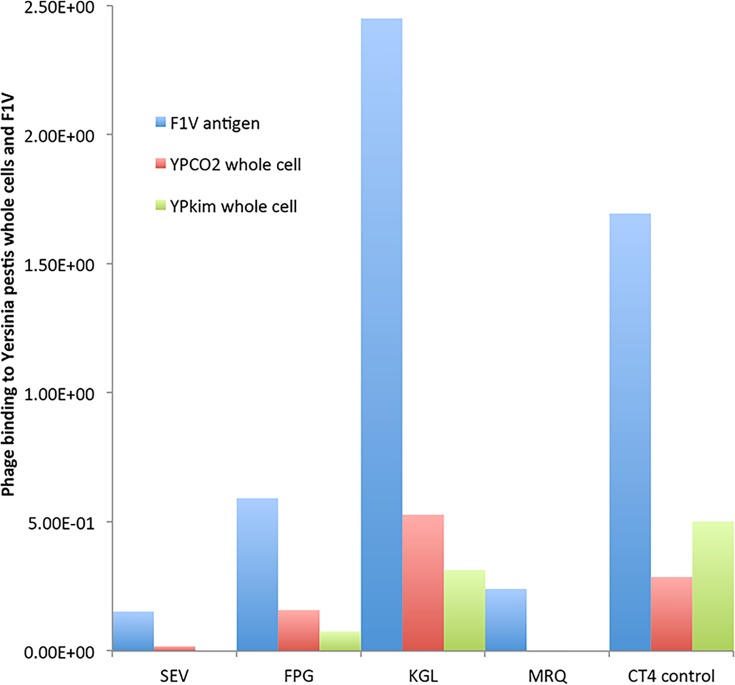
Two peptide clones recognize live whole cell *Y*. *pestis* as well as purified F1V antigen. Phage supernatants produced from clones randomly picked from selection outputs were assayed for binding to F1V-coated, whole cell-coated and uncoated wells. CT4 control is an scFv identified as positive from a previous selection on F1V [23]. Phage binding was detected with anti-M13-HRP conjugated antibody. The signal obtained with uncoated wells was subtracted from the signal corresponding to coated wells, and the results were normalized based on phage concentration.

Libraries were selected on purified F1V antigen, which was biotinylated and immobilized on streptavidin magnetic beads. After three rounds of selection, approximately 150 clones per selection output were assayed for binding to F1V antigen, by phage ELISA. The 48 clones with the highest signal/background ratio in ELISA (approximately 20-fold over background) were also sequenced (Table 2). It is interesting to note that a large number of the 48 F1V-positive clones contain a hydrophobic amino acid at the N-terminus. Surprisingly, none of the cyclic peptide libraries yielded ELISA positive clones, whereas all the linear libraries did. Repeated ELISA assays and re-sequencing of the F1V-ELISA positive clones revealed an astonishing consensus across the different libraries. The VxVN sequence was found among the majority of the clones analyzed, regardless of length, while the consensus FxVRVN was found among the 6 and 12-mers. Some of the F1V-ELISA-positive clones were further analyzed by whole-cell ELISA using *Y*. *pestis* C092 and *Y*. *pestis* Kim (Fig 4). Among the clones analyzed by whole-cell ELISA, the ones positive by both antigen and whole-cell ELISA were two 12-mers: FSVRVNLGPKGL (“KGL”), which conformed to our observed FxVRVN consensus, and FLQLHNLWPFPG (“FPG”), which conformed to our observed FxxxxN consensus (KGL and FPG are boxed in Table 2). It is worth noting here that two clones positive against *Y*. *pestis* whole cells (out of 48 clones positive for F1V) seems to be quite a low number. Several factors could be responsible for this result. First, we used phage culture supernatants for both types of ELISA assays. These supernatants typically produced variable numbers of phage and likely, given the typical affinity of peptides, may not have sufficient ligand concentration for detection of whole cells by ELISA (i.e., supernatants whose titer was less than 1x10^7^ pfu per milliliter were negative even by F1V ELISA). Second, ELISA on F1V differs from that on *Y*. *pestis* whole cells in that the whole cells secrete most of the F1 and V proteins into the surrounding medium, whereby most of it is rinsed off before encountering our phage clones [22].

**Table 2:** Consensus sequences in peptides selected from linear libraries against purified F1V antigen.

Selected peptides																																															
6-mers						12-mers															18-mers																										
W	F	V	R	V	N			E	W	R	V	N	V	N	G	R	M	R	Q								W	D	S	W	L	S	L	L	G	R	R	G	R	E	R	L	P	I			
F	W	V	R	V	N				F	R	V	G	V	N	R	I	S	H	W	A										W	K	A	R	V	N	L	T	D	V	H	R	N	Q	P	L	R	L
W	L	A	K	V	N				F	R	V	N	V	N	S	T	T	L	A	T							A	E	R	W	A	V	R	V	N	G	Q	P	V	A	V	R	W	L			
W	R	P	R	V	N				F	R	V	R	V	N	Q	A	G	S	E	V						E	A	G	R	W	V	V	R	V	N	G	G	A	I	L	S	N	R				
F	K	V	R	V	N				F	R	V	S	V	N	V	Q	E	T	P	R								R	W	F	A	M	P	V	D	S	R	W	I	P	Y	L	G	K	G		
F	L	V	R	V	N	F	S	H	F	V	V	R	V	N	N	M	R									G	L	A	G	W	A	S	W	P	R	T	R	G	T	F	A	S	I				
F	R	V	L	V	N				F	S	V	G	V	N	H	R	I	R	P	M							P	A	W	W	H	G	L	F	A	L	Q	M	E	P	T	R	Q	R			
F	R	V	K	V	N				F	S	V	R	V	N	L	G	P	K	G	L	P	R	M	E	Q	A	L	M	R	F	M	V	R	V	N	D	Q	P									
F	R	V	R	V	N				F	T	V	K	T	N	G	V	A	I	L	E										F	S	V	N	V	N	R	P	H	G	A	R	W	P	Q	P	V	V
F	R	V	S	V	N				F	T	V	K	T	N	G	Y	P	P	T	P					N	A	A	G	G	W	T	V	L	V	N	V	K	A	R	P	S	S					
F	T	V	R	V	N				F	V	V	K	V	N	Q	T	F	Q	A	S				K	P	R	Y	E	R	W	V	A	K	V	N	H	R	L	S	P	P						
F	V	V	R	V	N				F	F	V	S	V	N	H	P	K	V	L	R				R	D	R	I	A	H	F	A	V	Q	V	N	Q	V	R	L	G	K						
								H	W	Q	A	F	V	N	Q	R	G	W	R																												
							P	K	F	V	V	R	V	N	G	L	G	S																													
						R	P	R	W	V	A	F	V	N	H	R	P																														
									W	S	P	R	V	N	M	K	Q	H	G	L																											
									W	S	V	F	V	N	R	P	M	Q	S	F																											
									F	G	V	R	V	N	M	G	R	Y	V	Q																											
									F	H	V	R	V	N	H	Q	R	A	Q	T																											
									F	L	Q	L	H	N	L	W	P	F	P	G																											
									F	L	V	T	V	N	S	P	Q	P	R	S																											
									F	M	V	S	V	N	P	R	Y	R	A	P																											
							F	R	F	A	V	K	V	N	R	S	P	A																													
									F	R	I	R	V	N	L	P	A	K	V	S																											
**F**	**R**	**V**	**R**	**V**	**N**				**F**		**V**	**R**	**V**	**N**																**W**		**V**		**V**	**N**												

Yellow = conserved amino acids

Other colors = highly represented amino acids with similar features (green = hydrophobic; blue = hydrophilic and basic; magenta = hydrophilic and polar)

Boxed sequences were positive by both antigen and whole-cell ELISA

### Selection of QBP-binding Peptides

**Fig 5:**
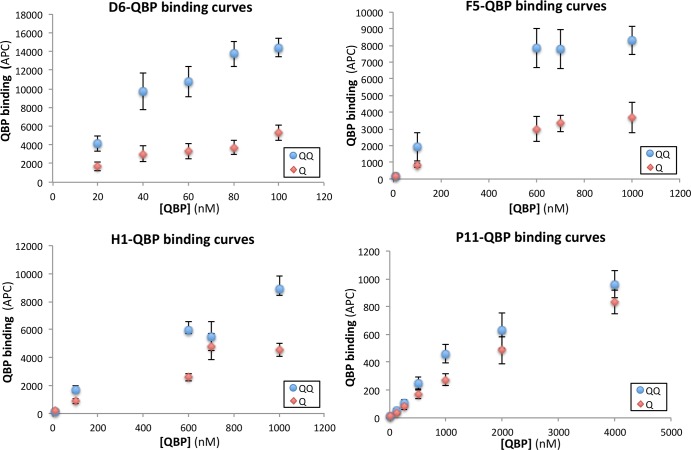
Binding curves for selected QBP-binding peptides. Selected peptides were yeast-expressed and assayed by flow cytometry for binding to apo (Q) or glutamine-bound (QQ) L-glutamine-binding periplasmic protein QBP at various concentrations. Experiments were executed in duplicate and the data points in each graph are averages of the mean APC fluorescence with corresponding standard deviations. The estimated affinities from this data are D6 = 35 nanoM, F5 = 300 nanoM, and P11 = 2 microM.

An equally represented mixture of primary phage libraries (3x10^12^ each) displaying 6, 10 and 12-mer peptides (one linear and two cyclic), was amplified in OminMAX M13cp-CT, and selected for binding to purified apo (Q) or glutamine-bound (QQ) L-glutamine-binding periplasmic protein QBP [20]. The sub-libraries of clones obtained at the end of the second round of phage panning in helper cells were cloned into the N terminus of the Aga-2 display protein in the yeast display vector, pCTCON2 [13]. The diversity of the two yeast display sub-libraries was approximately 10^7^. Among the members of these sub-libraries, those exhibiting the highest level of expression, and binding to either QQ or Q, were subjected to two rounds of selection by flow cytometry sorting. Among the output yeast from the second sort, forty-eight clones (one set per sort) were randomly picked and assayed for binding to the protein against which they were selected. 84% of the clones selected for binding to Q and 54.5% of the clones selected for binding to QQ were positive. The eleven best ligands (10-fold or more above background) in each set were tested for binding to either Q, QQ or streptavidin (no antigen control), in order to: 1) identify ligands capable of differentiating between glutamine-bound and unbound QBP; and 2) identify ligands that bind streptavidin. The eleven best clones sorted for binding to Q were confirmed as being Q-ligands, however one of them (D6) showed preferential binding to QQ vs Q (Fig 5), and none bound streptavidin. The eleven best clones sorted for binding to QQ were confirmed as QQ-ligands, however one (F5) bound QQ better than Q (Fig 5), and one bound streptavidin. Genes encoding peptide D6 and F5 (differential ligands), peptide P11 and H1 (binding equally well to Q and QQ, Fig 5), and the only streptavidin-binding peptide were sequenced and translated (Table 3). The QBP-binding peptides revealed a remarkable consensus, in particular the two stronger differential ligands bear more resemblance to each other (consensus: xDWxWMWPxF) than to the other two peptides (consensus: SKWSxMWRxx). In contrast to the F1V selections, here the best ligands were cyclic (nonlinear) 10-mers, while the streptavidin ligand was a linear 12-mer containing the motif GDW found in streptavidin peptides previously reported [24]. The GDW and DW motifs are also present in F5 and D6 respectively; these motifs might be related to streptavidin binding. Note that streptavidin is always present in our phage or yeast-based selections; therefore there might be a bias toward streptavidin binding for all the sequences that we select. It is possible that F5 and D6 are very weak streptavidin binders, but much stronger QQ and Q binders, whereas F4, for which the motif GDW is in a context different than for F5 and D6, streptavidin binding prevails.

**Table 3:** Sequence analysis of selected QBP or streptavidin-binding peptides.

		TARGET BOUND	SEQUENCE^a^											
**Selected peptides**	P11111	QQ and Q	C	S	K	W	S	W	M	W	R	D	Y	C
	H11	QQ and Q	C	S	K	W	S	G	M	W	R	A	F	C
**Consensus sequence**		QQ and Q	C	S	K	W	S	X	M	W	R	X	X	C
**Selected peptides**	D6	QQ > Q	C	Q	D	W	D	W	M	W	P	V	F	C
	F5	QQ > Q	C	G	D	W	G	W	M	W	P	A	F	C
**Consensus sequence**		QQ > Q	C	X	D	W	X	W	M	W	P	X	F	C
**Selected peptide**	F4	Streptavidin	**G**	**D**	**W**	G	A	W	V	V	S	G	G	R

^a^ Amino acids highlighted in color are conserved in multiple selected peptides.

Orange = small hydroxyl; blue = basic; green: hydrophobic; red: acidic; grey = cysteins needed for cyclization.

## Discussion

The helper plasmid-based system was developed as an efficient method to create libraries that are free of contaminating helper phage while having tunable display levels. In this manuscript we first characterize and optimize the helper plasmid system for peptide display, and then demonstrate its utility in selecting relevant peptides against two different targets. A careful analysis of transformation efficiency, susceptibility to phage infection, and efficiency of phage production of several combinations of helper plasmids and *E*. *coli* strains (S1 Fig), has lead us to conclude that: 1) peptide libraries can be cloned directly in all the helper cells derived from *E*. *coli* SS320, with an efficiency of transformation comparable to the parent cell line; 2) OmniMAX-M13cp-CT and DH5α-M13-DG3 can be infected by phage as efficiently as *E*. *coli* XL1Blue (our standard of “infectability”), and are high-yield phage producers. *E*. *coli* SS320 have the highest transformation efficiency of any strain used for phage display (≥4x10^10^ cfu/microgram pUC vector), so it is not surprising that the helper cells derived from this strain are also very efficient phage producers (S1 Fig). OmniMAX-M13cp-CT had the highest susceptibility to phage infection, while OmniMAX-DG3 was consistently found resistant to phage infection. It is unclear why the F’ OmniMAX cells would be susceptible to phage infection while carrying the M13-cp-CT plasmid, yet resist infection while carrying the M13-DG3 plasmid. However, for this reason we did not choose OmniMAX-DG3 helper cells for further studies.

We created high diversity, high quality linear and cyclic primary peptide libraries of variable lengths directly in helper cells SS320-M13cp (Table F in S1 File). Upon selection against F1V [16–19] and L-glutamine-binding periplasmic protein QBP [20], these libraries yielded positive linear (F1V or streptavidin ligands) or cyclic (QBP-ligands) peptides of variable lengths, bearing different consensus sequences for different target ligands (Tables 1 and 2). Upon screening of phage sub-libraries obtained from panning against F1V, peptides FPG and KGL were found to be the best ligands, as they demonstrate reproducible binding to F1V antigen, as well as to live *Y*. *pestis* CO92 and KIM (Fig 4). The similarity of results obtained in the two different assays, and the vast [FxVRVN] consensus within KGL and the like, indicates that these peptides are true F1 ligands.

Our selection of QBP-binding peptides combined two rounds of phage library panning in OmniMAX-M13cp-CT, with two subsequent rounds of panning on yeast. Such toggling between display scaffolds is particularly useful since small molecules like peptides are more prone than are larger proteins toward bias derived from display context. The high percentage of positive clones randomly picked from the second round of yeast selection against free QBP (Q) or glutamine-bound QBP (QQ), demonstrates the high efficiency of our selection method. We were able to find peptides capable of distinguishing between bound and unbound QBP, mostly likely due to the large total number of positive clones. The sizes of the QBP or Streptavidin-binding peptides were consistent with the size range of our libraries (Table 3, 10-mer cyclic QBP ligands and 12-mer linear Streptavidin ligand) suggesting that there were no truncations during selections. Furthermore the four QBP-binding clones revealed a remarkable sequence similarity, which is consistent with their similar function. It is interesting to notice that the two peptides (D6 and F5) with higher affinity for QQ than for Q have higher sequence similarity than the two peptides that bind equally to both forms of QBP. Although streptavidin was not a molecule that we targeted for selection, a streptavidin-binding peptide containing the tripeptide GWD, previously reported as a consensus sequence for other streptavidin-binding peptides [24] was selected, further validating the value of our libraries.

Based on the results presented in this manuscript we can confidently state that a comprehensive array of peptide libraries can be produced and selected in our optimized helper cell system, leading to the identification of peptides of various lengths and structures, which bind to ligands of interest.

## Materials and Methods

### Helper cells preparation and characterization

#### Helper cells preparation

The three helper plasmids used in the construction of helper cells were M13cp, M13cp-CT, and M13-DG3. Cell lines DH5alpha, SS320 (made in house by mating MC1061 from ATCC with XL1Blue from Stratagene), and OmniMaxT1 (Invitrogen), were made chemically competent with MgCl_2_ and CaCl_2_ and transformed with the helper plasmids by heat shock, followed by plating on 2XYT containing 20 microgram/milliliter chloramphenicol (2XYT/cam). The nine resulting transformants (helper cell lines) were proven to contain the correct construct by PCR using primers Gene 6 (5’-CTCGAAAGCAAGCTGATAAACCGATACA-3’) and Gene 8 (5’-CCCAAAAGAACTGGCATGATTAAGACTCC-3’). Every helper cell clone tested revealed the presence of the correct length amplicon (M13cp = 1366 bp, M13cp-CT = 613 bp, and M13-DG3 = 236 bp).

#### Transformation efficiency

The helper cell lines were made electrocompetent, and transformed with pDAN5-D1.3 phagemid [11]. Dilutions of the transformed cells were plated on 2XYT containing 100 microgram/milliliter carbenicillin (2XYT/carb) and counted. Transformation efficiency was reported as the number of colony-forming units per microgram of DNA.

#### Infectability (susceptibility to infection)

The helper cell lines were grown overnight in 2XYT/cam, diluted 100-fold in 50 milliliter 2XYT/cam, and grown to mid-log phase (OD600 0.5). Cells were infected with purified pDAN5-D1.3 phage particles which were Polyethylene glycol-sodium chloride (PEG-NaCl)-purified and stored at -80°C in 15% glycerol and thawed just before use) at multiplicity of infection (MOI) between 100 and 1000 MOI. Cells were infected for 30 minutes in a stationary 37°C incubator, then allowed to recover by incubation at 37°C with shaking (250 rpm) for 60 minutes. After the recovery, 100 microliter aliquots were taken from the cell cultures, serially diluted, and plated on either 2XYT/cam or 2XYT/carb. Infectability was calculated as the percent of carb-resistant colonies in the total number of cam-resistant colonies, *i*.*e*., the percent of helper cells that were infected by phage.

#### Phage production

The remaining 49.9 milliliter of infected helper cell cultures from the infectability studies were used in the phage production studies. Cells were centrifuged at 2800 rpm at 18°C for 30 minutes to remove excess D1.3 input phage. The cells were re-suspended in 2XYT containing 100 microgram/milliliter carbenicillin and 10 microgram/milliliter chloramphenicol (2XYT/carb/cam), and grown overnight at 30°C with shaking at 250 rpm. BL21 (Promega) cells were also infected as a negative control. BL21 are non-infectable because they are missing the F1 pilus, so they were included in order to determine the level of phage background in our phage preparations. After 19 hours, the cells were pelleted at 4000 rpm (4°C) for 60 minutes, and the supernatants were titrated to determine the amounts of phage produced by each helper cell line. Phage production was calculated as the total number of amplified phage particles relative to the phage obtained from the BL21 culture (background), and reported as fold-amplification over background.

### Library preparation and characterization

#### Generation of random peptide DNA libraries by PCR

The phagemid pDAN5-D1.3 was used as the template for large-scale (10 milliL) PCR reactions to generate each DNA library. In each PCR various sets of primers incorporated random peptide sequences. The primers were from Trilink Biotechnologies (San Diego, CA). The antisense primer, “StAantsns”, which primed in the PelB region of pDAN5D1.3, had the sequence 5’GTGATGGGTTAAAAAGCGCGCCGCTTGCTG-3’, and was used to make all ten libraries. Each sense primer annealed to 5’ region of gene 3 while incorporating a BssHII restriction site, a His-Ala-Ala spacer and a random peptide of various length. Each amino acid of the random peptide was coded by the NNK codon. The general sequence of the sense primers used for the linear peptide libraries construction was:

5’-AACGTGTGCGCGCATGCAGCC(NNK)_x_GCCGCTACTGTTGAAAGTTGTTTA-3’, where X was either 6, 12 or 18 for the 6, 12, or 18-mer library respectively. The general sequence of the sense primers used for the cyclic peptide libraries (also 6, 12, or 18-mer) construction was: 5’-AACGTGTGCGCGCATGCAGCCGGCTGT(NNK)_x_TGTGGTGCCGCTACTGTTGAAAGTTGTTTA-3’. These primers had the same features as the primers used for the linear libraries; additionally, they were designed so that each side of the random peptide would be flanked by a glycine and a cysteine. PCR was performed using Phusion taq polymerase as follows: 0.2 micromolar each of dNTP and primers, 100 units Phusion taq, and 3 microgram DNA template in a final volume of 10 milliL. The thermocycle program was: 98°C 30 sec, (98°C 10 sec, 68°C 20 sec, 72°C 90 sec) x30, 72°C 10 min. PCR product was cleaned with QIAQuick PCR cleanup kit (Qiagen). Cleaned PCR products were digested with BssHII, purified by agarose gel electrophoresis (QIAexII, Qiagen) and circularized with T4 DNA ligase. The ligation reactions were cleaned by ethanol precipitation. All enzymes were from New England Biolabs (Beverly, MA).

#### Generation of primary phage libraries in SS320-M13cp cells

DNA from all five random peptide libraries was transformed into our helper cell line SS320-M13cp. Although helper cells have a lower efficiency of transformation than standard *E*. *coli* SS320, the goal was to create the libraries in helper cells in order that the primary phage libraries would be free of helper phage. The transformation procedure was the following: 2 milliliter of cells were electroporated in ten cuvettes (200 microliter cells per cuvette) with DNA library (between 500 nanog and 10 microgram DNA library, depending on yield from DNA purification). Cultures were pooled, Super-Optimal broth plus glucose (SOC) was added to a total of 20 milliL, and the cultures were shaken at 37°C for 30 minutes. Subsequently, 80 more milliliter of SOC was added, and the cultures were shaken at 37°C for 30 minutes. Small aliquots were removed for library size determination, while the remainder of the cultures was added to 900 milliliter of 2XYT/carb/cam to make primary phage libraries. Library sizes were calculated by: 1) plating dilutions of the transformed cells on 2XYT/carb/cam; 2) counting antibiotic-resistant colonies; 3) extrapolating the total number of colony-forming units in each library. The amplified primary phage libraries were PEG/NaCl precipitated, titrated in DH5alpha, and stored at -80°C as glycerol stocks.

#### Generation of secondary libraries in Omni-M13cp-CT and XL-DG3 cells

Each of the 5 primary phage libraries was used to make secondary phage libraries by infecting either our Omnimax-M13cp-CT helper cells (monovalent display) or our XL-DG3 helper cells (multivalent display). One liter of cell culture was infected with primary phage at MOI of less than 1, and incubated with shaking overnight at 30°C. Secondary phage libraries were PEG/NaCl precipitated twice, titrated in DH5alpha and stored as glycerol stocks at -80°C.

#### Sequencing of unselected secondary libraries

After spotting 5 microliter of infected DH5α on 2XYT/carb plates for titer, 100 microliter of the higher dilutions (10^−9^, 10^−10^, 10^−11^) cell suspensions were plated on 2XYT/carb plates for single colony growth. Approximately 200 to 400 colonies were randomly picked and sequenced from each library. Sequencing FW primer was 5’-GCA CGA CAG GTT TCC CGA CT-3’.

### Library Selections

#### Selection and screening of F1V ligands

Antigen-conjugated beads for selection on F1V surface protein were prepared as follows: 1 millig of *Yersinia pestis* fusion protein F1V (BEI) was coupled to 20 millig of Dynal MyOne carboxylic acid magnetic beads using *N*-hydroxysulfosuccinimide/1-ethyl-3-(3-dimethylaminopropyl) carbodiimide hydrochloride (NHS/EDC) chemistry. Conjugation of the protein to the beads was performed in 23 millimolar sulfo-NHS and 26 millimolar EDC, (Pierce) in 25 millimolar 2-(*N*-morpholino)ethanesulfonic acid (MES) buffer pH 6 for 1h at room temperature, followed by quenching in 50 millimolar Tris-HCl pH 7.5 for 15 minutes and washing twice with 1X Phosphate-buffered saline (PBS). The beads were re-suspended to 10 millig/milliliter in PBS for use in selections. F1V protein conjugated to the beads was quantified by BCA assay (Pierce) and was determined to be 2.14 millig/milliliter (2.14 millig F1V to 10^10^ beads). Prior to selection the ten random peptide phage display secondary libraries prepared as described above were blocked with 4.5% bovine serum albumin (BSA) in a 200 microliter volume for 1h at room temperature with rotation. At the same time, a volume of F1V-conjugated bead solution corresponding to 6 micromolar F1V was aliquoted in as many Eppendorf tubes as number of selections desired, and blocked with 4.5% (BSA) under the same conditions as the blocked phage. The beads were collected using a Dynal magnetic rack and re-suspended in the blocked phage solutions. The mixture of phage and beads was then incubated together in an automated magnetic bead system (KingFisher,) for one hour, followed by six ten-second washes with 200 microliter PBS + 0.5% BSA to remove unbound phage. Finally, the bound phage was eluted from the beads in 100 microliter of 0.1M HCl for 15 minutes, and neutralized by adding 50 microliter of 1.7 M Tris-HCl pH 8. The eluted phage was amplified by infecting 4 milliliter of XL-DG3 cells grown to OD600 0.5 with 130 microliter of phage for 60 minutes at 37°C, followed by the addition of 21 milliliter of 2XYT/carb/cam, and incubated overnight at 30°C with shaking. The supernatants from the overnight cultures were PEG/NaCl precipitated once and re-suspended in PBS with protease inhibitor (Roche) and PMSF. The amplified phage outputs were subjected to two subsequent rounds of selection, whose stringency was increased by decreasing the concentration of bead-bound F1V to 3.5 micromolar (second round) and 2.2 micromolar (third round). The phage output solutions obtained from the third round of each selection were screened for F1V binding by ELISA. Helper cell lines infected with the phage outputs were plated on 2XYT/carb/cam at a low enough density to obtain single colonies. The next day, 96 colonies per output were picked into 800 microliter of 2XYT/carb/cam in sterile deep 96-well plates, and incubated overnight at 30°C for phage production. Concurrently, NUNC maxisorp plates were prepared as follows: rows A, C, E, G received 100 microliter per well of a 10 microgram/milliliter F1V protein solution in PBS, while rows B, D, F, H received only 100 microliter of PBS (no antigen negative control). The maxisorp plates were incubated overnight at 4°C. ELISA was carried out by washing the coated plates once with PBS and blocking with 250 microliter of 4.5% fish gelatin in PBS at room temperature (RT) for 1h. During the blocking, 700 microliter of infected XL-DG3 cells (after 100 microliter of cell suspension was put aside for sequencing) were pelleted in a deep well plate at 4000 rpm at 4°C for 45 minutes, in order to obtain phage supernatants for ELISA. Upon removing the blocking solution, 25 microliter of 4.5% fish gelatin was added to each well of the ELISA plates, along with 75 microliter of each phage supernatant. The plates were incubated for 1.5 hours at room temperature, then washed twice with 0.1% Tween-20 in PBS (PBST) and then twice with PBS. Upon addition of 100 microliter of 3.7 microgram/milliliter rabbit-anti-M13 IgG (Abcam) and 1% fish gelatin in PBS, the plates were incubated for one hour at room temperature, then washed twice with PBST and twice with PBS. This was followed by addition of 100 microliter of 2 microgram/milliliter goat-anti-rabbit IgG-HRP (Abcam) in 0.45% fish gelatin. The plates were incubated for 45 minutes at room temperature, washed twice with PBST and twice with PBS. The plates were developed by adding 100 microliter TMB substrate (Pierce), followed by addition of 50 microliter of 1 M H_2_S0_4_. The plate was read at 405 nm on a Thermo Multiskan Ascent spectrometer. Positive clones (clones 10-fold or more over background) were sequenced, and the phage ELISA was then repeated for all positive clones.

#### Whole-cell phage ELISA

Phage supernatants were prepared in deep-well plates as described above. Sterile NUNC maxisorp plates were coated overnight with *Yersinia pestis* strain C092 or Kim as follows: rows A, C, E, G were coated with 100 microliter per well of *Yersinia* in 100 microliter Tryptic Soy Broth (TSB). The remaining row received only TSB. In addition to the anti-F1V peptide phage clones, a previously analyzed anti-F1V scFv phage, CT4 [23], was used as the positive control and an F1V-negative phage was used as the negative control. The whole cell ELISAs were carried out using the protocol used for antigen ELISA. Signals corresponding to cell-free wells were subtracted from signal corresponding to coated wells. The resultant numbers were normalized based on phage titers, and the final results were reported as folds above negative phage binding.

#### Selection and screening of QBP-binding peptides by phage display

**Phage production:** Helper cell *E*. *coli* OmniMAX M13cp-CT was cultured in 10 milliliter 2XYT plus 20 microgram/milliliter chloramphenicol (2XYT/cam), to OD600 0.7, and infected with an equally represented mixture of phage libraries displaying 6, 10 and 12-mer peptides (linear and cyclic) at MOI 100, by static incubation at 37°C for 45 min. The cell suspension was centrifuged, re-suspended in 50 milliliter 2XYT/carb/cam and incubated at 30°C overnight. The cell suspension was centrifuged, after which the supernatant was filter sterilized and PEG-NaCl precipitated twice. The second precipitate was re-suspended in 500 microliter PBS, and titrated in *E*. *coli* DH5alpha.

**Biotinylation of QBP:** A 12-fold excess of Sulfo-NHS-LC-LC-Biotin (PIERCE) was added to a 78 micromolar protein (either QBP or QQ, obtained by incubating QBP with 26-fold molar excess of L-glutamine at 30°C for 30 min) solution in PBS. Solutions were kept on ice for 2 h, and dialyzed in PBS (QBP) or PBS plus 2 millimolar L-glutamine (QQ). Biotinylation level was quantified using the Pierce Biotin Quantitation Kit according to the vendor instructions. Biotinylation levels varied between 1:1 to 1:2 protein:biotin.

**Panning:** The amplified phage display library mixture (190 microL, 2x10^12^ cfu, obtained as described above) was pre-blocked by adding 10 microliter of WonderBlock (WB: PBS, 0.3% Skim milk, 0.3% fish gelatin, 0.3% BSA), and incubating at RT for 30 min. 30 microliter streptavidin-bound magnetic beads (Invitrogen) were washed four times in 300 microliter PBST, blocked by incubation in WB for 20 min at RT, washed 3x in PBST and re-suspended in 600 microliter PBST. 1 microliter of either biotinylated QQ, QBP (prepared as described above), or PBS (negative control) was added to the blocked phage. 200 microliter of phage with biotinylated antigen or PBS were added to the first row of a 96-well KingFisher plate, whereas three 200 microliter aliquots of blocked beads (plus 2 millimolar glutamine for libraries incubated with QQ) were added to the second row. The subsequent 2 rows contained 200 microliter of PBST, the next 3 contained 1X PBS, 0.01% tween-20 (PBSLT) or PBSLT plus 2 millimolar glutamine (for libraries incubated with QQ), and the last row contained 150 microliter 0.1 M HCl. Phage antigen incubation occurred with slow mixing and lasted 1 h in the first and second cycle of panning, and 0.5 h in the third cycle. The sample containing phage, antigen, and beads was incubated with slow mixing for 15 min. The washes occurred with medium mixing speed, and lasted 5, 15 and 30 min in the first, second and third cycle of panning respectively. Elution occurred at medium mixing speed, and lasted 3 min, after which 50 microliter of Tris-HCl/HCl 1 M pH 8 was added for neutralization, and beads were removed. Half (100 microliter) of the eluted phage from each well was stored as glycerol stock, and the other half was incubated with 2 milliliter *E*. *coli* Omnimax M13cp-CT helper cells (OD600 0.7) for 46 min at 37°C. 10 microliter infected cells were used for phage titration, while the rest was plated on 2XYT/cam/carb and incubated overnight at 30°C. Infected cells were scraped the day after in 2 milliliter 2XYT, and partially used to seed 50 milliliter 2XYT/cam/carb at OD600 0.1. After 2 h incubation at 37°C with shaking (250 rpm), cultures were incubated overnight at 30°C with shaking. The remainder of the scraped infected cells from each round of panning was stored as a glycerol stock at -80°C. Amplified phage was purified and concentrated as described above, and used for the next round of selection.

**Yeast display sub-library preparation. Insert preparation:** Phage stored at -80°C after each selection cycle, or phage used as input in the first panning cycle, was used to infect *E*. *coli* DH5alpha F’ (OD600 = 0.7), by incubation at MOI 2 in 1 milliliter 2XYT, at 37°C for 45 min. Infected cells were plated on 2XYT/carb, and incubated at 37°C overnight. Carb-resistant colonies were scraped and mini-prepped the day after. The plasmid preps were used as templates for the following polymerase chain reactions (PCR). The reaction mixture (330 microliter) contained Thermopol buffer 1x (NEB), 200 micromolar of each dNTP, 0.7 micromolar FW and REV primers (pDAN5topCON2FW: GGA GGC GGA GGG TCG GCT AGC AGC GGC GCG CAT GCG GCC and pDAN5topCON2REV: AAT AAG CTT TTG TTC GGA TCC ACT TTC AAC AGT AGC GGC, from MWG Operon), 50 units/milliliter Taq polymerase (NEB) and 33 nanogram plasmid. Thermocycling conditions were: 95°C 5 min, (95°C 30 sec, 60°C 20 sec, 72°C 45 sec) x 30 cycles, 72°C 7 min. Products of the correct size were gel purified.

**Vector preparation:** pTCON2 vector was obtained from the Wittrup lab [13]. Upon cloning and amplification in DH5alpha F’ followed by mini-prepping, the plasmid was restriction digested (BamHI, NheII and SalI) and gel purified.

**Competent yeast preparation:** *S*. *cereviseae* EBY100 from a fresh agar plate was grown overnight in yeast extract peptone dextrose (YPD), at 30°C, with shaking (250 rpm). Cultures were diluted in pre-warmed fresh YPD medium to a cell density of 5x10^6^ cells/milliliter (OD600 0.13) and incubated at 30°C with shaking (250 rpm) until cell density increased by an order of magnitude (about 7 h). Yeast were harvested by centrifugation (3500 rpm for 5 min), re‐suspended in half the initial volume of water, and centrifuged again. This step was repeated once, followed by re-suspension in 1 milliliter Transformation Buffer (Sigma). Competent cells were stored overnight at 4°C.

**Yeast transformation:** Competent yeasts were pelleted at max speed in an Eppendorf centrifuge for 15 s, and the supernatant was removed. Salmon sperm DNA (Life Technologies), DNA inserts, cut pTCON2, and plating buffer (Sigma) were added in the order indicated, followed by incubation at 30°C for 45 min, heat shock at 42°C for 1h, cell pelleting, removal of supernatant, and re-suspension in water. Efficiency of transformation was determined by counting the number of colonies developed on solid selective medium (synthetic dextrose/casamino acid, plus 50 microgram/milliliter kanamycin and 15 microgram/milliliter tetracycline, or SD/CAA). The size of the libraries was extrapolated from this count. Libraries were amplified by diluting the transformants in 100 milliliter SD/CAA to OD600 0.4, and incubated at 30°C until appropriate cell density (OD600 5.0) was reached (about 3 days). Library sizes were 1–0.5x10^7^ transformants, with 10–4% background. Amplified libraries were stored as 30% glycerol stocks, containing approximately 10^8^ total cells.

#### Selection and screening of QBP-binding peptides by yeast sorting

**Yeast expression:** Frozen library stocks were diluted in 1 milliliter SD/CAA to OD600 0.2, and grown overnight at 30°C. Overnight cultures were diluted to OD600 0.25 in induction medium (synthetic galactose-raffinose/casamino acid, plus 50 microgram/milliliter kanamycin and 15 microgram/milliliter tetracycline, or SGR/CAA) and grown at 20°C, with shaking (250 rpm), for 20–72 hours.

**Yeast sorting:** Induced yeast (300 microliter) were split into two wells of an ultrafiltration plate (Millipore), washed 3x with yeast washing buffer (YWB, 0.5% BSA, 2 millimolar EDTA), re-suspended in a YWB solution containing 0.5 microgram/milliliter mouse anti c-myc primary antibody (Invitrogen), 100 nanomolar Biotinylated QBP, QQ (plus 1 micromolar glutamine) or lysozyme (negative control), and incubated at RT with shaking (8000 rpm) for 30 min. Cells were washed 3x with YWB (plus 1 μM glutamine for libraries selected on QQ), re-suspended in a YWB solution containing streptavidin Alexa_633_ (Molecular Probes) and goat anti-mouse-PE (Abcam), and incubated at RT for 30 min with shaking. Cells were washed 3x with YWB and re-suspended in 1 milliliter of YWB, followed by flow cytometry analysis and sorting (BD-FACSAria). Sorted cells were collected in 100 microliter YPD medium, amplified in SD/CAA solid medium, scraped (part of the scrapes were stored as 30% glycerol stocks), induced (as described above), and subjected to the next round of selection and sorting.

**Single yeast clone binding assay:** Approximately 1x10^3^ cells from the glycerol stocks of the sorted libraries were plated on fresh SD-CAA plates. Well-spaced single clones were picked in 100 microliter SD-CAA medium, aliquoted in a 96-well round bottom plate (NUNC), and grown overnight at 30°C. 15 microliter of overnight culture was inoculated into 100 microliter of SR-CAA (aliquoted in the wells of a 96-well ultrafiltration plate), and incubated at 20°C with shaking (10000 rpm). The rest of the overnight cultures were stored as 30% glycerol stocks (by adding 100 microliter 60% glycerol) at -80°C. Induced yeast cultures were treated as described above for the libraries and analyzed by high throughput flow cytometry (BD LSRII). See an example of analysis in S2 Fig.

**Sequencing:** Single clones were analyzed by sequencing (MWG operon) using T7 promoter and T7 terminator standard primers (for genes in pDAN5).

#### ScFv library construction, selection, and screening

The primary, secondary, and tertiary helper phage scFv antibody libraries were constructed as described earlier [11]. The helper plasmid libraries were similarly constructed; however, phage was produced using helper plasmids [7] instead of M13K07 helper phage. Plasmid DNA was made from the primary library [11] and was transformed into electrocompetent SS320 bacteria containing the M13cp helper plasmid, followed by phage production. The resulting primary helper plasmid library was recombined by infecting bacteria expressing cre recombinase [11, 25] containing the M13cp-CT helper plasmid, followed by helper plasmid phage production. Finally, the tertiary helper plasmid library was generated by infecting the secondary helper plasmid phage into M13cp-CT DH5alpha F’ bacteria and subsequent phage production. Three rounds of phage selections were performed using either the tertiary phage antibody library prepared using either helper phage (M13K07) or the helper plasmid (M13cp-CT) against a biotinylated lysozyme target. Dilution plates between rounds were plated, single clones picked, and PCR amplified to determine the number of full length scFvs (Table A in S1 File). Full length clones from the third round selection outputs were tested by phage ELISA. ELISA positive clones were BSTN1 fingerprinted to determine the number of unique scFvs (Table B in S1 File). Infection of secondary helper plasmid library into m13cp-CT was plated on either Amp or Amp/Cap plates. Single colonies were picked, grown for phage production, and PCR amplified to verify full length scFvs (Table C in S1 File). A series of tertiary libraries was amplified using either helper plasmids (M13cp-CT) or helper phage (M13K07). Phage production was determined by titration and single colonies were picked and PCR amplified to verify full length scFvs. The lowest and highest numbers of phage produced and percent full length scFvs were determined (Table D in S1 File). Plates were made with ampicillin and decreasing concentrations of chloramphenicol. After infection of a helper plasmid secondary phage antibody library into M13cp-CT, the infections were plated on each type of plate. Dilution plates were used to determine infection rates and single colonies were picked and PCR amplified to verify full length scFvs. The remaining M13cp-CT phage producing bacteria were scraped and phage production was determined by titration.

## Supporting Information

S1 Fig Helper cells characterization The transformation efficiency (colony forming units, cfu/amount of DNA used for transformation) of helper cells derived from *E*. *coli* SS320, OmniMAX or DH5alpha F’relative to *E*. *coli* SS32 (non helper gold standard) was determined in the same experiment. All the helper cells derived from *E*. *coli* SS320 exhibited higher transformation efficiencies than the other helper cells. Helper cells and *E*. *coli* XL1 Blue (non helper gold standard) were infected in identical conditions with phage displaying the single chain antibody fragment scFv D1.3. Infectability was expressed as percentage of transformants relatively to *E*. *coli* XL1 Blue. As previously discussed, Omnimax-DG3 was not infected at all, whereas both the *E*. *coli* SS320-derived helper cells tested were 50% as efficiently infected as *E*. *coli* XL1 Blue. OmniMAX-CPCT, DH5alpha-DG3 and XL1 Blue DG3 were as infectable as *E*. *coli* XL1 Blue. The most highly infectable cell lines were also tested for efficiency of phage production. Phage production was tested on a 50 milliliter scale, in identical conditions. Phage released in the supernatant was titrated, and the total number of phage particles (colony-forming units, pfu) in 50 milliliter supernatant was calculated. The most efficient phage producers, OmniMAX-CPCT and XL1 Blue-DG3, were chosen for generating our secondary libraries (with intermediate and high display respectively).
(EPS)

S2 Fig Flow cytometry analysis of yeast expressing peptide F5. (A) Entire yeast population detected by forward and side scatter (FSC and SSC respectively). (B) Expressing (main peak) and non-expressing (minor peak) fractions of yeast population, detected with anti-SV5 antibody conjugated to phycoerythrin (PE). (C) Yeast binding (main peak) or non-binding (minor peak) to biotinylated, glutamine-bound PBP, as detected with Alexa_632_-conjugated to streptavidin (APC). (D) The expressing and binding yeast population (carrying APC as well as PE fluorescence) is shown in green.
(TIF)

S1 File Supplementary tables. Comparison of the percent of full length scFvs during phage selection against biotinylated lysozyme (Table A). Comparison of the number of full length scFvs during phage selection against biotinylated lysozyme (Table B). Effect of dual selective pressure on the amplification of a tertiary helper plasmid scFv library (Table C). Comparison helper phage scFv library to helper plasmid scFv library (Table D). Effect of chloramphenicol concentration on: infection titer, phage production, and full length scFvs (Table E). Peptide libraries characterization based on transformation efficiency and sequencing (Table F).
(DOCX)
