# Supplementary figures and images for: Beyond Helper Phage: Using "Helper Cells" to Select Peptide Affinity Ligands
Source: PLoS One. 2016 Sep 14;11(9):e0160940. doi: 10.1371/journal.pone.0160940 (PMC5023105; doi:10.1371/journal.pone.0160940)

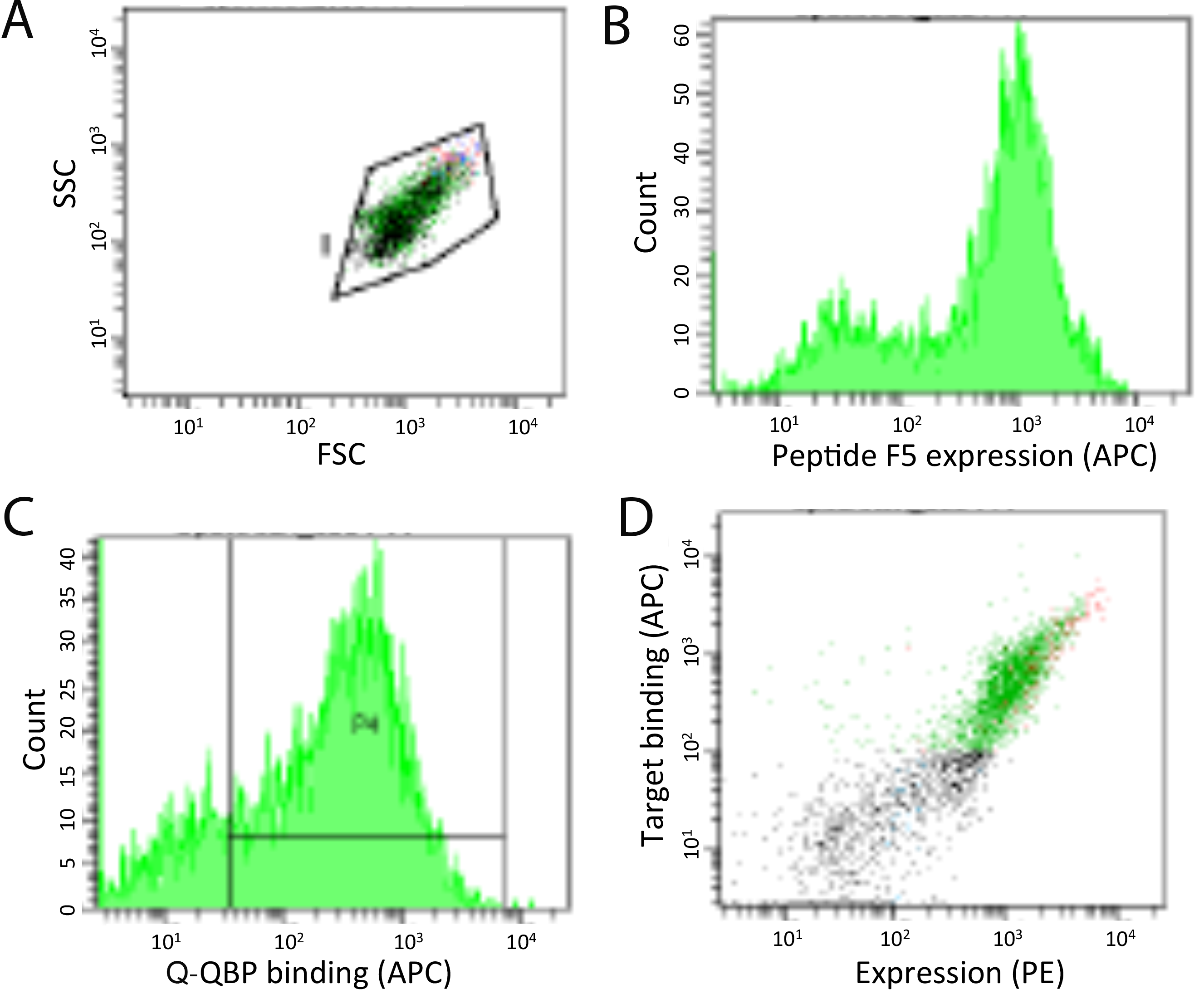

Supplement: S2 Fig — (A) Entire yeast population detected by forward and side scatter (FSC and SSC respectively). (B) Expressing (main peak) and non-expressing (minor peak) fractions of yeast population, detected with anti-SV5 antibody conjugated to phycoerythrin (PE). (C) Yeast binding (main peak) or non-binding (minor peak) to biotinylated, glutamine-bound PBP, as detected with Alexa632-conjugated to streptavidin (APC). (D) The expressing and binding yeast population (carrying APC as well as PE fluorescence) is shown in green. (TIF) [file pone.0160940.s002.tif]
