# Supplementary material for: Beyond Helper Phage: Using "Helper Cells" to Select Peptide Affinity Ligands
Source: PLoS One. 2016 Sep 14;11(9):e0160940. doi: 10.1371/journal.pone.0160940 (PMC5023105; doi:10.1371/journal.pone.0160940)
Supplement: S1 File — Comparison of the percent of full length scFvs during phage selection against biotinylated lysozyme (Table A). Comparison of the number of full length scFvs during phage selection against biotinylated lysozyme (Table B). Effect of dual selective pressure on the amplification of a tertiary helper plasmid scFv library (Table C). Comparison helper phage scFv library to helper plasmid scFv library (Table D). Effect of chloramphenicol concentration on: infection titer, phage production, and full length scFvs (Table E). Peptide libraries characterization based on transformation efficiency and sequencing (Table F). (DOCX) [file pone.0160940.s003.docx]

**Supplementary Tables:**

**Table A.** **Comparison of the percent of full length scFvs during phage selection against biotinylated lysozyme.**

| Library | % full length  after 1^st^ round | % full length after 2^nd^ round | % full length after 3^rd^ round |
| --- | --- | --- | --- |
| M13KO7 | 63 | 78 | 93 |
| M13cp-CT | 50 | 28 | 14 |

Conclusion: Phage produced with M13K07 result in an increase in full length scFvs between rounds of phage selection while phage produced with m13cp-CT result in a decrease.

**Table B.** **Comparison of the percent of full length scFvs during phage selection against biotinylated lysozyme.**

| Library | Number of phage ELISA positives | Number of full length positive scFvs | Number of unique full length positive scFvs |
| --- | --- | --- | --- |
| M13KO7 | 27 | 25 | 3 |
| M13cp-CT | 17 | 5 | 4 |

Conclusion: Although M13cp-CT tertiary phage libraries have half the number of full length scFv and continue to lose full length scFv clones between rounds of selection, the resulting number of full length positive clones are almost all unique. Furthermore, the antibodies selected using M13cp-CT are different to those selected using M13K07.

**Table C.** **Effect of dual selective pressure on the amplification of a tertiary helper plasmid scFv library**.

|  | % colonies that produce phage | % colonies that are full length |
| --- | --- | --- |
| Amp plate | 49 | 78 |
| Amp/CAP plate | 100 | 45 |

Conclusion: in order to ensure that phage particles are produced, both ampicillin and chloramphenicol selection pressure must be applied to ensure that both phagemid (ampicillin) and the helper plasmid (chloramphenicol) are retained. However, this results in a reduction in the number of colonies that contain phagemid clones that are full length.

**Table D**. **Comparison helper phage scFv library to helper plasmid scFv library.**

| Tertiary library produced by: | Range of phage production/ml | Range of % full length scFv’s |
| --- | --- | --- |
| M13cp-CT | 5 X 10^10-11^ | 45 - 65 |
| M13KO7 | 1 X 10^12-13^ | 85 - 95 |

Conclusion: Amplified helper plasmid libraries (M13cp-CT) have a 10-fold reduction in phage production and contain approximately half the number of full length scFvs compared to tertiary libraries produced using helper phage (M13K07).

**Table E.** **Effect of chloramphenicol concentration on: infection titer, phage production, and full length scFvs.**

| Concentration of CAP in plates (ug/ml) | Infection titer/ml | Phage production titer/ml | % full length scFv’s |
| --- | --- | --- | --- |
| 20 | 5 X 10^8^ | 6 X 10^11^ | 40 |
| 10 | 3 X 10^8^ | 6 X 10^11^ | 45 |
| 5 | 2 X 10^8^ | 3 X 10^11^ | 50 |
| 0 | 1 X 10^8^ | 1 X 10^11^ | 70 |

Conclusion: Chloramphenicol selective pressure is necessary for phage production using helper plasmids, but results in a reduction of full length phagemid clones. Reducing chloramphenicol selective pressure, while slightly decreases phage infection and phage production rates, does not result in an significant increase in the number of full length phagemid clones.

**Table F. Peptide libraries characterization based on transformation efficiency and sequencing.**

| **Library** | **Theoretical**  **diversity**^a^ | **Actual diversity**  **of primary library**^b^ | - **CPCT secondary library** | | - **DG3 secondary library** | |
| --- | --- | --- | --- | --- | --- | --- |
|  |  |  | - % truncations^c^ | - % out-of-frame^d^ | - % truncations^c^ | - % out-of-frame^d^ |
| - 6-mer L | 6.4E+7 | 9.3E+07 | - 0 | - 0 | - 0.1 | - 1.9 |
| - 12-mer L | 4.1E+15 | 1.9E+08 | - 2.8 | - 3.5 | - 0 | - 2.0 |
| - 18-mer L | 2.6E+23 | 1.2E+09 | - 1.1 | - 3.3 | - 0.9 | - 1.8 |
| - 6-mer C | 6.4E+7 | 4.0E+08 | - 0 | - 0 | - 0 | - 1.1 |
| - 10-mer C | 1.0E+13 | 4.9E+08 | - 1.3 | - 0 | - 7.1 | - 2.3 |
| - ^a^ 20^x^, X = peptides length - ^b^ number of single clones obtained by transformation of helper cells SS320-CP - ^c ,d^ # truncated or out of frame clones*100/total number of clones sequenced | | | | | | |
